# Bacterial coinfection and antibiotic resistance in hospitalized COVID-19 patients: a systematic review and meta-analysis

**DOI:** 10.7717/peerj.15265

**Published:** 2023-04-26

**Authors:** Ruhana Che Yusof, Mohd Noor Norhayati, Yacob Mohd Azman

**Affiliations:** 1 Department of Medicine, Faculty of Medicine, Universiti Malaya, Kuala Lumpur, Wilayah Persekutuan Kuala Lumpur, Malaysia; 2 Department of Family Medicine, Universiti Sains Malaysia, Kubang Kerian, Kelantan, Malaysia; 3 Medical Development Division, Ministry of Health, Putrajaya, Malaysia

**Keywords:** Bacterial coinfection, COVID-19, Antibiotic-resistant bacteria, Prevalence, Systematic review

## Abstract

**Background:**

There were a few studies on bacterial coinfection in hospitalized COVID-19 patients worldwide. This systematic review aimed to provide the pooled prevalence of bacterial coinfection from published studies from 2020 to 2022.

**Methods:**

Three databases were used to search the studies, and 49 studies from 2,451 identified studies involving 212,605 COVID-19 patients were included in this review.

**Results:**

The random-effects inverse-variance model determined that the pooled prevalence of bacterial coinfection in hospitalized COVID-19 patients was 26.84% (95% CI [23.85–29.83]). The pooled prevalence of isolated bacteria for *Acinetobacter baumannii* was 23.25% (95% CI [19.27–27.24]), *Escherichia coli* was 10.51% (95% CI [8.90–12.12]), *Klebsiella pneumoniae* was 15.24% (95% CI [7.84–22.64]), *Pseudomonas aeruginosa* was 11.09% (95% CI [8.92–13.27]) and *Staphylococcus aureus* (11.59% (95% CI [9.71–13.46])). Meanwhile, the pooled prevalence of antibiotic-resistant bacteria for extended-spectrum beta-lactamases producing Enterobacteriaceae was 15.24% (95% CI [7.84–22.64]) followed by carbapenem-resistant *Acinetobacter baumannii* (14.55% (95% CI [9.59–19.52%])), carbapenem-resistant *Pseudomonas aeruginosa* (6.95% (95% CI [2.61–11.29])), methicillin-resistant *Staphylococcus aureus* (5.05% (95% CI [3.49–6.60])), carbapenem-resistant Enterobacteriaceae (4.95% (95% CI [3.10–6.79])), and vancomycin-resistant Enterococcus (1.26% (95% CI [0.46–2.05])).

**Conclusion:**

All the prevalences were considered as low. However, effective management and prevention of the infection should be considered since these coinfections have a bad impact on the morbidity and mortality of patients.

## Introduction

The severe acute respiratory syndrome coronavirus-2 (SARS-CoV-2, 2019-nCoV) is a novel coronavirus that causes the disease known as coronavirus disease 2019 (COVID-19), which primarily affects the respiratory system (Yuki, Fujiogi & Koutsogiannaki, 2020), and the majority of viral respiratory tract infections are linked with microbial coinfection (Langford et al., 2020). Coinfection with a virus, bacteria, or fungus significantly impacts how COVID-19 infection develops and spreads, complicating COVID-19 diagnosis, treatment, and prognosis and even worsening disease symptoms and mortality (Chen et al., 2020).

A previous meta-analysis involving 20 published studies from December 2019 to June 2021 estimated the pooled prevalence of bacterial coinfection in patients with COVID-19 was 5.62% (95% CI [2.26–10.31]) (Alshaikh et al., 2022). The coinfections frequently occurred in patients hospitalized for an extended period, aggravated respiratory viral infections, and frequently resulted in fatalities during influenza pandemics (Westblade, Simon & Satlin, 2021). It was similar for COVID-19 patients in a multicenter retrospective cohort study in the United States, where the coinfections were more common in critically ill COVID-19 patients (Langford et al., 2020), particularly those who required invasive mechanical ventilation (Petty et al., 2022).

The most common bacteria isolated from co-infected COVID-19 patients were *Streptococcus pneumoniae*, *Staphylococcus aureus*, and *Haemophilus influenzae* (Moreno-García et al., 2022). Furthermore, a review reported that the most frequent bacteria were coagulase-negative *Staphylococcus* isolated from blood, *Streptococcus pneumoniae* from the respiratory tract, and *Escherichia coli* from urinary tract samples (Santos et al., 2022). Another review (Abu-Rub et al., 2021) evaluated the presence of bacteria such as *Klebsiella pneumonia, Pseudomonas aeruginosa*, and *Acinetobacter baumannii* co-infected in an intensive care unit (ICU) COVID-19 patients.

According to literature analysis, patients with COVID-19 admitted to the ICU had an unexpectedly high frequency of infections caused by methicillin-resistant *Staphylococcus aureus* (MRSA), carbapenem-resistant *Acinetobacter baumannii* (CRAB), carbapenem-resistant Enterobacteriaceae (CRE), and *Candida auris* (Segala et al., 2021). Meanwhile, a review discovered that over half of ICU patients with SARS-CoV-2 infection were prescribed antibiotics, significantly higher than the estimated frequency of identified bacterial co-infection (Abu-Rub et al., 2021). The review also stated that the WHO’s guidance on the clinical management of COVID-19 alarmed that antibiotic overuse increases the risk of multidrug-resistant organisms (MDROs) emerging and spreading.

This review aimed to reveal the pooled prevalence of bacterial coinfection and antibiotic resistance in hospitalized COVID-19 patients. This information integrates existing information to determine if scientific conclusions are consistent and generalizable across populations and situations or whether findings significantly differ by subgroup. It also aids healthcare shareholders in making decisions because the pooled prevalence increases the power and precision of effect estimations. It also included the prevalence of isolated bacteria related to bacterial coinfection and the prevalence of antibiotic resistance related to bacterial coinfection. However, only certain bacteria and selected antibiotic resistance bacteria were involved in this review.

## Materials and Methods

### Types of studies

The pooled prevalence of bacterial coinfection, pooled prevalence of certain bacteria (*Acinetobacter baumannii, Escherichia coli, Klebsiella pneumoniae, Pseudomonas aeruginosa* and *Staphylococcus aureus*) isolated from patients with positive bacterial coinfection and the pooled prevalence of antibiotic resistance bacteria (CRE, CRAB, CRPA, VRE, MRSA and ESBL‑producing Enterobacteriaceae) among hospitalized COVID-19 patients was assessed using a systematic review and meta-analysis. A further analysis based on the year of publication, regions, and wards was done as subgroup analyses for the bacterial coinfection.

This review was registered in the PROSPERO database (CRD42022364268). Before its submission, formal screening of search results against eligibility criteria was performed to ensure the measurement of interest outcomes was compatible with the study objectives. The review is acceptable if they have not advanced beyond the point of performing data extraction. Preliminary searches and piloting of the study selection process also were done together in this step. Data extraction for this review was started after the registration. The institutional review board statement was not applicable. Ethics review and approval are not required for analyses of published data.

### Search methods

Between January 2020 and September 2022, a systematic search for related articles was conducted in three databases: MEDLINE (PubMed), Web of Sciences, and Scopus. The search was done using four keyword concepts of prevalence, bacterial coinfection, antibiotic resistance, and hospitalized COVID-19. The combination of the Medical Subject Headings (MeSH) and text words such as “((prevalence [MeSH] OR prevalence [Text Word] OR proportion [Text Word]) AND (bacterial coinfection [MeSH] OR “bacterial coinfection” [Text Word])) AND (antibiotic resistance [MeSH] OR “antibiotic resistance” [Text Word] OR “antimicrobial resistance” [Text Word]) AND (COVID-19 [MeSH] OR “COVID-19” [Text Word] OR “coronavirus” [MeSH] OR “coronavirus” [Text Word] OR “SARS-COV-2” [MeSH] OR “SARS-COV-2” [Text Word])” were used for the searching process.

Different electronic databases could employ the search terms. All studies released between January 2020 and September 2022 were retrieved in order to determine whether they qualified for inclusion in this research. English full-text articles only were included in the search results. In order to find additional potential eligible studies, secondary citations from the articles were included for cross-checking.

### Study selection

All records discovered through the research method were saved in EndNote software. Duplicate articles have been removed. Two independent reviewers (RCY and MNN) screened the titles and abstracts of the identified articles. If the two reviewers disagreed, a consensus was convened and a third reviewer (MYA) was consulted. The PRISMA flow chart depicted the search method, which included studies and those excluded, as well as the reasons for exclusion.

### Inclusion and exclusion criteria

The full texts of eligible studies were retrieved and thoroughly read to determine eligibility. This study excluded case reports, conference papers, proceedings, articles available only in abstract form, editorial reviews, letters of communication, commentaries, reviews, and qualitative studies. This study also excluded animal studies and genomic studies. Articles unrelated to COVID-19 patients, no coinfection, out-of-interest outcomes, and incomplete data were excluded from this review. Only studies with complete data that fulfilled the criteria of the interested outcomes were selected to include in this study.

### Data extraction and management

The relevant details were entered into Microsoft Excel. Name of the first author, year of publication, study site, design, population, sample size, setting, type of bacterial infection (bloodstream infection (BSI), secondary infection, and bacterial superinfection), the total number of isolations, type of bacteria and its number of isolated, type of antibiotic-resistant bacteria and its number of isolated, and data used to calculate effect estimates were all included.

### Assessment risk of bias

The Joanna Briggs Institute (JBI) critical appraisal checklist for studies reporting prevalence was used to assess the data quality (Munn et al., 2020). Bias was evaluated independently by two authors. When more than 70% of the responses were “yes”, the risk of bias was deemed low, moderate when 50% to 69% of the responses were “yes”, and high when 0 to 49% of the responses were “yes”. Reviews of studies with a high or moderate risk of bias would be disregarded (da Silva Rosa et al., 2020).

### Measures of outcome

The main purpose of this review was to determine the pooled prevalence of bacterial coinfection in hospitalized COVID-19 patients. The COVID-19 patients were confirmed with reverse transcription polymerase chain reaction (RT-PCR) test. This review also included bloodstream infection (BSI), secondary bacterial infection, and bacterial superinfection, considered bacterial coinfection in this study. Bacterial coinfection is an infection that develops within 48 h after a positive COVID-19 diagnosis and being admitted to the hospital (Alshaikh et al., 2022). Meanwhile, only BSI developed 48 h after admission (secondary infection) were included. Secondary infections were recognized as bacterial infections that emerged during ICU stay but after admission of more than 48 h, implying that they were not present during the COVID-19 presentation. Superinfections are commonly used to describe secondary bacterial infections in COVID-19 patients (Pourajam et al., 2022). Apart from that, this review also pooled the prevalence of isolated bacteria and the prevalence of antibiotic-resistant bacteria.

The prevalence of bacteria coinfection was calculated as the total number of patients with positive specimens (for at least one bacteria) divided by the total number of patients involved in the study. The subgroup analyses for this outcome were the year, regions, and wards.

The prevalence of bacteria isolated from the patients with positive bacterial coinfection was calculated as the total number of the isolated bacteria divided by the total number of bacteria isolation. Bacteria involved in this review were *Acinetobacter baumannii*, *Escherichia coli*, *Klebsiella pneumoniae*, *Pseudomonas aeruginosa*, and *Staphylococcus aureus*.

Meanwhile, the prevalence of antibiotic-resistant bacteria was calculated as the total number of antibiotic resistance bacteria divided by the total number of bacteria isolated. The selected antibiotic resistance bacteria were carbapenem-resistant Enterobacteriaceae (CRE), carbapenem-resistant *Acinetobacter baumannii* (CRAB), carbapenem-resistant *Pseudomonas aeruginosa* (CRPA), vancomycin-resistant Enterococcus (VRE), methicillin-resistant *Staphylococcus aureus* (MRSA) and extended-spectrum beta-lactamases (ESBL) producing Enterobacteriaceae.

### Data synthesis

A pooled prevalence estimates with a 95% confidence interval (CI) served as the prevalence. Software Review Manager version 5.4 was used to conduct the analysis (Nordic Cochrane Centre). The DerSimonian-Laird estimate of tau^2^ was used to pool the data using the random-effects inverse-variance model. The following guidelines were followed when evaluating heterogeneity using the I^2^ statistic: 0% to 40% might not be significant, 30% to 60% might be moderate heterogeneity, 50% to 90% might be significant heterogeneity, and 75% to 100% could represent considerable heterogeneity (Higgins et al., 2022). Visual analysis of the funnel plot and Egger’s test statistics were used to determine whether there was a chance of publication bias. Stata 13.1 test (StataCorp, Texas) was used to determine Egger’s. Subgroup analyses were performed based on year (2020, 2021, and 2022), wards (ICU and unspecific wards), and regions (Africa, America, Asia, and Europe).

## Results

### Study selection

**Figure 1:**
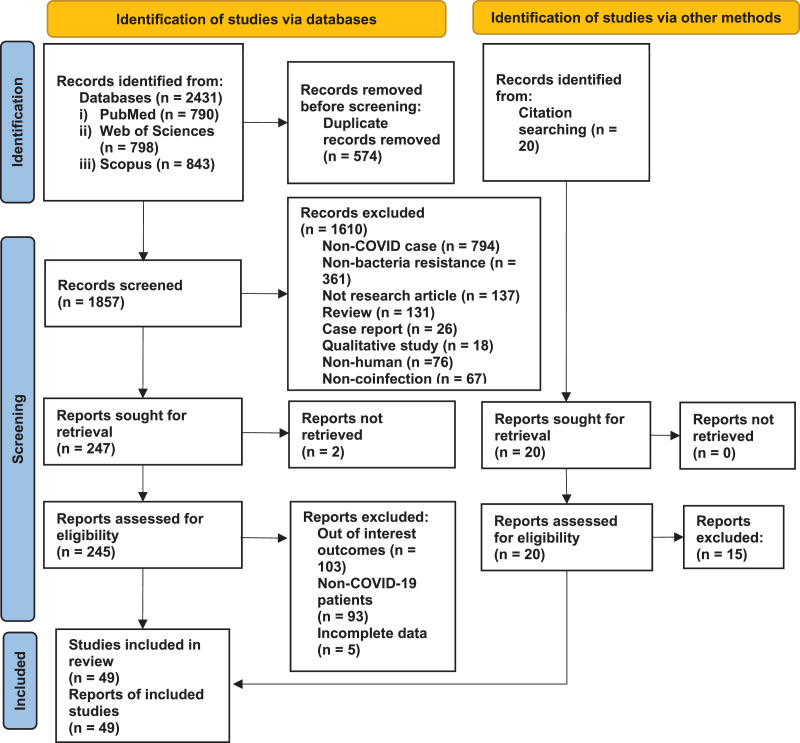
PRISMA flow chart.

Databases and secondary citations yielded a total of 2,451 studies. Titles and abstracts screened were 1,857 after removing duplicate studies. Then, 265 studies were retrieved from databases and secondary citations and evaluated for eligibility. In this review, only 49 studies that matched the inclusion and exclusion criteria were considered (Fig. 1).

### Study characteristics

The included studies were published from 2020 to 2022 from regions of Africa (Egypt) (Ramadan et al., 2020), America (United States, Peru, and Brazil) (Baggs et al., 2022; Copaja-Corzo et al., 2021; da Costa et al., 2022; Kubin et al., 2021; Nori et al., 2021), Asia (Pakistan, Saudi Arabia, Qatar, India, Iran, Iraq, China, Indonesia, and Korea) (Ahmed et al., 2022; Alqahtani et al., 2022; Baiou et al., 2021; Bazaid et al., 2022; Bhaskaran, 2022; Boorgula et al., 2022; Jabir, 2022; Jamnani et al., 2022; Khurana et al., 2021; Li et al., 2020; Mahmoudi, 2020; Moradi et al., 2021; Palanisamy et al., 2021; Pourajam et al., 2022; Rao et al., 2022; Rizvi et al., 2022; Said et al., 2022; Sathyakamala, Peace & Shanmugam, 2022; Sharifipour et al., 2020; Sharma et al., 2021; Sinto et al., 2022; Son et al., 2021; Vijay et al., 2021; Zeshan et al., 2022) and Europe (Turkey, United Kingdoms, Italy, France, Czech Republic, Switzerland, Germany, Greece, and Spain) (Bahce, Acer & Ozudogru, 2022; Baskaran et al., 2021; Bentivegna et al., 2021; Ceccarelli et al., 2022; Contou et al., 2020; Cultrera et al., 2021; Doubravska et al., 2022; Floridia et al., 2022; Fontana et al., 2021; Foschi et al., 2021; Gysin et al., 2021; Karataş et al., 2021; Karruli et al., 2021; Lingscheid et al., 2022; Mazzariol et al., 2021; Posteraro et al., 2021; Protonotariou et al., 2021; Suarez-de-la-Rica et al., 2021; Temperoni, Caiazzo & Barchiesi, 2021). Three studies were designed as cross-sectional studies (Mahmoudi, 2020; Moradi et al., 2021; Said et al., 2022), 10 studies were prospective studies (Gysin et al., 2021; Jabir, 2022; Lingscheid et al., 2022; Mazzariol et al., 2021; Ramadan et al., 2020; Rizvi et al., 2022; Sharifipour et al., 2020; Sharma et al., 2021; Zeshan et al., 2022), and the remaining 36 studies were retrospective studies. Twenty-four studies involved COVID-19 patients from ICU; the rest were from unspecific wards that combined ICU and non-ICU wards. The specimens for the culture were from blood, respiratory tract secretions, or urinary samples.

### Risk of bias assessment

Data quality assessment for risk of bias showed that all studies were low risk of bias with “yes” scores from seven to nine over nine criteria listed in the checklist (Table S1).

### Prevalence of bacterial coinfection

Overall, the pooled prevalence of bacterial coinfection in hospitalized COVID-19 patients was 26.84% (95% CI [23.85–29.83]). The heterogeneity of data by a random effects model was considerable (I^2^ > 99%). Subgroup analysis based on the publication year showed that the prevalence of bacterial coinfection was highest in the year 2022 (34.26%; 95% CI [24.78–43.74]) compared to the previous years. The second subgroup analysis by wards showed that the COVID-19 patients in ICU had a higher prevalence of bacterial coinfection than unspecific wards. Meanwhile, the region’s subgroup analysis determined that the prevalence of bacterial coinfection was highest in Europe region (39.19%; 95% CI [30.47–47.91]) compared to other regions. The details were showed in Table 1.

**Table 1:** The prevalence of bacterial coinfection in the hospitalized COVID-19 patients.

Outcome	No. of studies	No. of participants	Prevalence (95% CI)	I² (%)	*p*-value
Bacterial coinfection	49	212,605	26.84 [23.85–29.83]	99	*p* < 0.001
Subgroup year					
2020	5	218	12.98 [7.46–18.49]	90	*p* < 0.001
2021	23	3,106	23.59 [20.74–26.44]	99	*p* < 0.001
2022	21	209,281	34.26 [24.78–43.74]	100	*p* < 0.001
Subgroup wards					
ICU	24	2,274	41.46 [29.95–52.96]	99	*p* < 0.001
Unspecific wards	25	210,331	18.87 [16.23–21.51]	99	*p* < 0.001
Subgroup regions					
Africa	1	28	10.77 [7.01–14.53]	NA	
America	5	207,257	22.04 [10.94–33.15]	99	*p* < 0.001
Asia	24	3,660	23.79 [19.41–28.16]	100	*p* < 0.001
Europe	19	1,660	39.19 [30.47–47.91]	99	*p* < 0.001

### Prevalence of isolated bacteria

The prevalence of bacteria isolated in hospitalized COVID-19 patients was showed in Table 2. The highest prevalence was *Acinetobacter baumannii* (23.25%; 95% CI [19.27–27.24]), and the lowest prevalence was *Escherichia coli* (10.51%; 95% CI [8.90–12.12]). The overall pooled prevalence from the five isolated bacteria was 9.65% (95% CI [9.28–10.02]). The heterogeneities for these analyses were high (I^2^ > 98%). The random effects model considers the differences between studies.

**Table 2:** The prevalence of bacterial isolated in the hospitalized COVID-19 patients.

Outcome	No. of studies	No. of isolations	Prevalence (95% CI)	I² (%)	*p*-value
Type of bacteria	49	21,622	9.65 [9.28–10.02]	98	*p* < 0.001
*Acinetobacter baumannii*	38	1,318	23.25 [19.27–27.24]	97	*p* < 0.001
*Escherichia coli*	40	7,226	10.51 [8.90–12.12]	94	*p* < 0.001
*Klebsiella pneumoniae*	42	4,130	19.18 [15.56–22.79]	98	*p* < 0.001
*Pseudomonas aeruginosa*	44	3,556	11.09 [8.92–13.27]	96	*p* < 0.001
*Staphylococcus aureus*	41	5,392	11.59 [9.71–13.46]	97	*p* < 0.001

### Prevalence of antibiotic-resistant bacteria

The pooled prevalence for the six antibiotic-resistant bacteria was 1.17% (95% CI [0.99–1.34]). The highest prevalence in this analysis was 15.24% (95% CI [7.84–22.64]) by the ESBL-producing Enterobacteriaceae from *Klebsiella pneumoniae* and *Escherichia coli*. The lowest prevalence was VRE, with 1.26% (95% CI [0.46–2.05]). CRAB, with a prevalence of 14.55% (95% CI [9.59–19.52]), was the highest prevalence among the other carbapenem-resistant bacteria. The heterogeneities of these random effects models were significantly high (I^2^ > 98%) (Table 3).

**Table 3:** The prevalence of antibiotic resistant bacteria in the hospitalized COVID-19 patients.

Outcome	No. of studies	No. of isolations	Prevalence (95% CI)	I² (%)	*p*-value
Antibiotic resistance bacteria	27	3,693	1.17 [0.99–1.34]	98	*p* < 0.001
MRSA	20	184	5.05 [3.49–6.60]	83	*p* < 0.001
ESBL-producing Enterobacterales	13	2,073	15.24 [7.84–22.64]	98	*p* < 0.001
CRAB	11	281	14.55 [9.59–19.52]	97	*p* < 0.001
CRE	17	270	4.95 [3.10–6.79]	91	*p* < 0.001
CRPA	9	381	6.95 [2.61–11.29]	86	*p* < 0.001
VRE	8	507	1.26 [0.46–2.05]	78	*p* < 0.001

**Note:**

MRSA, Methicillin-resistant *Staphylococcus aureus;* ESBL, extended-spectrum beta-lactamases producing Enterobacterales; CRAB, carbapenem-resistant *Acinetobacter baumannii*; CRE, carbapenem-resistant Enterobacteriaceae; CRPA, carbapenem-resistant *Pseudomonas aeruginosa*; VRE, vancomycin-resistant Enterococcus.

### Publication bias

**Figure 2:**
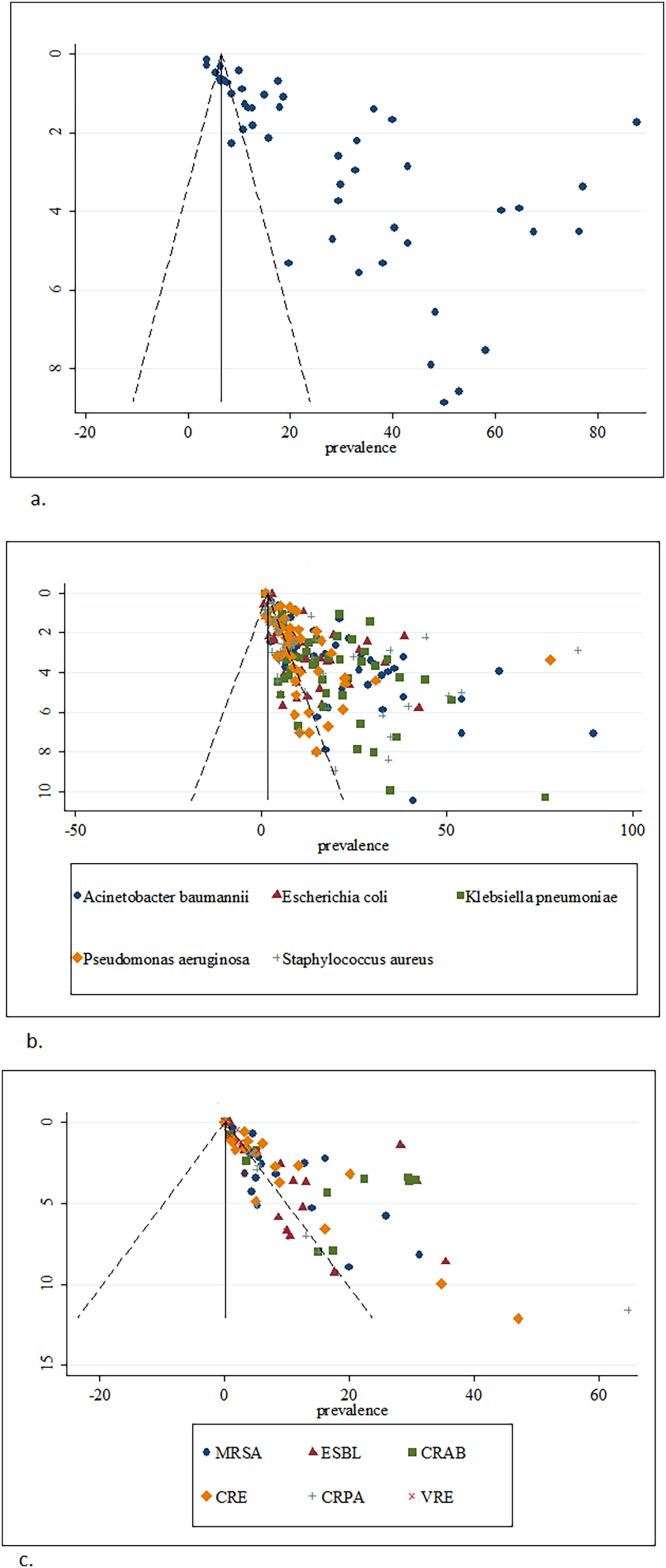
Funnel plots of (A) prevalence of bacterial coinfection, (B) prevalence of isolated bacteria in the bacterial coinfection and (C) prevalence of antibiotic resistant bacteria in the bacterial coinfection.

**Figure 3:**
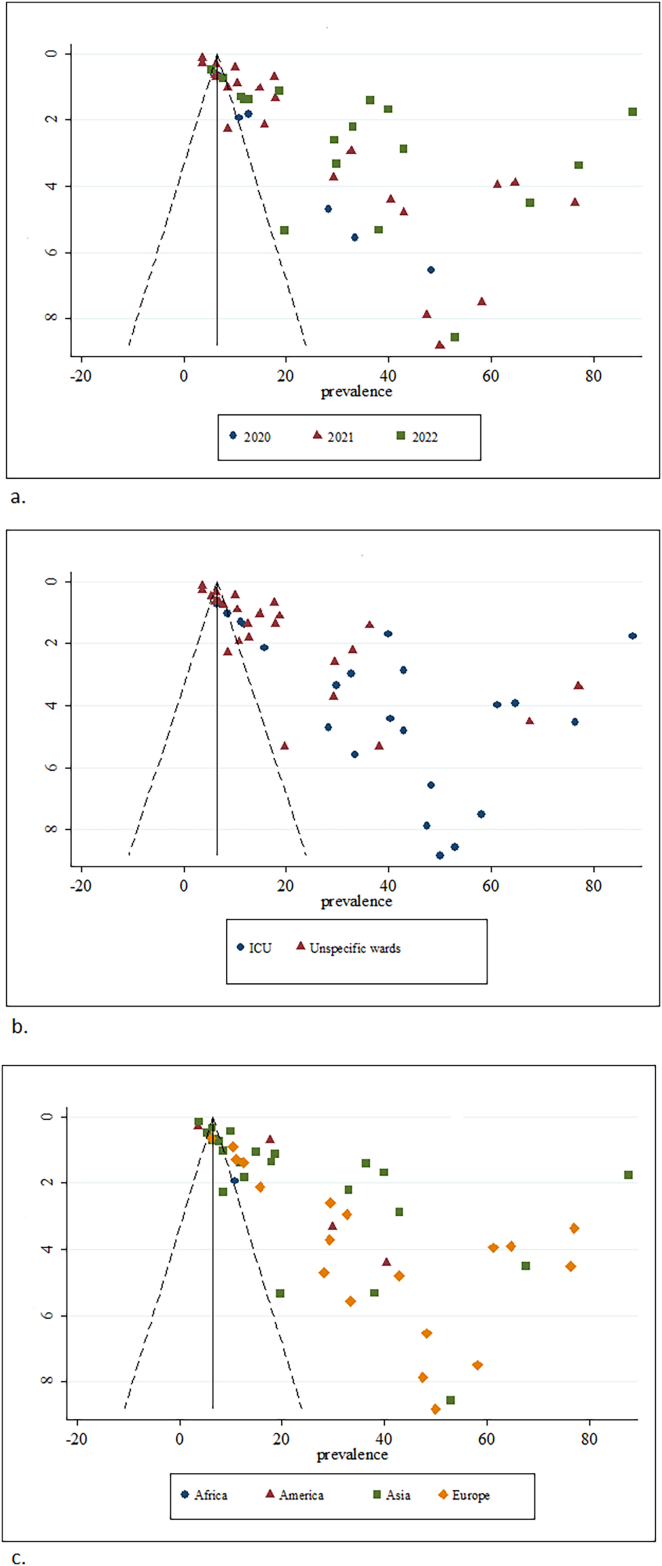
Funnel plot of prevalence of bacterial coinfection by subgroups of (A) year, (B) ward, and (C) region.

The funnel plots showed that the distributions were asymmetry for all plots (Figs. 2 and 3). The distributions were concentrated on the right side of the plots. Egger’s test proved that there were small studies effects by significant *p*-values for all variables (Table 4).

**Table 4:** Egger’s test.

Egger test	Coefficient	(95% CI)	*p*-value
Bacterial coinfection	11.21	[8.08–14.24]	<0.001
Subgroup year			
2020	5.57	[2.92–8.21]	0.007
2021	9.76	[7.02–12.50]	<0.001
2022	14.81	[5.76–23.86]	0.003
Subgroup wards			
ICU	10.89	[3.40–18.39]	0.007
Unspecific wards	10.67	[7.29–14.05]	<0.001
Subgroup regions			
Africa	NA		NA
America	12.37	[−16.19 to 40.94]	0.203
Asia	12.66	[6.89–18.42]	<0.001
Europe	10.74	[7.04–14.44]	<0.001
Type of bacteria			
*Acinetobacter baumannii*	6.11	[4.32–7.90]	<0.001
*Escherichia coli*	2.85	[1.83–3.86]	<0.001
*Klebsiella pneumoniae*	5.54	[4.18–6.89]	<0.001
*Pseudomonas aeruginosa*	3.75	[2.61–4.90]	<0.001
*Staphylococcus aureus*	3.40	[1.69–5.11]	<0.001
Antibiotic resistance bacteria			
Methicillin-resistant *Staphylococcus aureus*	2.49	[1.33–3.64]	<0.001
ESBL-producing Enterobacterales	4.20	[0.55–7.86]	0.028
Carbapenem-resistant *Acinetobacter baumannii*	4.50	[2.25–6.74]	0.001
Carbapenem-resistant Enterobacteriaceae	2.89	[2.00–3.79]	<0.001
Carbapenem-resistant *Pseudomonas aeruginosa*	2.49	[1.21–3.77]	0.003
Vancomycin-resistant Enterococcus	1.83	[0.67–2.99]	0.008

## Discussion

In this review of 49 studies, the pooled prevalence of bacterial coinfection in hospitalized COVID-19 patients was 26.84% (95% CI [23.85–29.83]). It was the highest compared to review studies published in 2020 (prevalence = 6.9% (95% CI [4.3–9.5])) (Langford et al., 2020) involving COVID-19 patients from 24 studies. Another review showed a prevalence of 7% (95% CI [3–12]) involving hospitalized COVID-19 patients from 30 studies (Lansbury et al., 2020). Meanwhile, a meta-analysis published in 2021 reported the prevalence of bacterial coinfection increased to 19% (95% CI [14–25]), and the prevalence of bacterial superinfection was 24% (95% CI [19–30]) (Musuuza et al., 2021) which pooled from 118 studies. Another meta-analysis in 2021 also reported the pooled estimated prevalence of bacterial coinfection in COVID-19 patients from 42 studies was 20.97% (95% CI [15.95–26.46]) (Soltani et al., 2021). The pooled prevalence of bacterial coinfection in COVID-19 patients for review of 25 studies published in 2022 was 24% (95% CI [8–40]) (Kariyawasam et al., 2022). There was an increasing pattern of prevalence from the year 2020 to 2022. Another factor that may influence the high prevalence of bacteria coinfection in this review was the consideration of bloodstream infection, secondary infection, and bacterial superinfection as bacterial coinfection.

A significantly increasing trend of bacterial coinfection prevalence was also shown by year subgroup analysis from 13% in 2020 to 24% in 2021 and higher increased in 2022 to 34% in this review. However, this situation might be influenced by the under-reporting of the incidence due to limitations of laboratory resources, especially during the early phase of the COVID-19 pandemic (Petty et al., 2022) or a limited number of studies investigating the coinfection (Jeong et al., 2022). The coinfection rate also differs due to geographical regions, study duration (Wee et al., 2020), variation in case definition (Baskaran et al., 2021), or differences in diagnostic approaches used for coinfection detection (Moreno-García et al., 2022).

Bacterial coinfection was higher in ICU COVID-19 patients compared to COVID-19 patients from unspecific wards. This result was similarly reported by a previous meta-analysis that compared ICU and non-ICU COVID-19 patients (Kariyawasam et al., 2022). According to a study from Saudi Arabia, ICU COVID-19 patients had higher levels of bacterial coinfection found in their blood and sputum, which may be related to their more extended stays in the ICU and increased use of catheters like endotracheal, arteriovenous, and urinary tubes (Bazaid et al., 2022). Another study (Soriano et al., 2021) discovered that ICU-acquired infections were high (51.2%), with the respiratory tract, bloodstream, urinary tract, soft tissue, and abdominal focus being the most common sites of infection.

Based on regions subgroup analysis, most of the studies were from Asia, with America having the largest number of participants, and the region’s highest prevalence of bacterial coinfection was Europe. In contrast, another review Alshaikh et al. (2022) found that America had the highest prevalence of bacterial coinfection, followed by Asia and Europe. According to the report, the prevalence may be influenced by potentially higher rates of microbial lab investigation and excessive interpretation of laboratory data. Additionally, variations in population, regions, and environment, access to care, and infection control and prevention practices may impact the frequency of bacterial coinfections (Langford et al., 2020).

In hospitalized COVID-19 patients, this review pooled the prevalence of isolated bacteria for *Acinetobacter baumannii* (23%), *Escherichia coli* (11%), *Klebsiella pneumoniae* (19%), *Pseudomonas aeruginosa* (11%) and *Staphylococcus aureus* (12%). All these bacteria were associated with nosocomial infection (Bazaid et al., 2022). Gram-negative was more prominent isolated in COVID-19 patients, and *Acinetobacter baumannii* has been identified as the primary pathogen in the patient’s respiratory tracts (Bazaid et al., 2022). *Acinetobacter baumannii* coinfection secondary to SARS-CoV-2 infections has been the subject of numerous reports, with an incidence rate of up to 1% of hospitalized COVID-19 patients from various regions (Rangel, Chagas & De-Simone, 2021). It has also been reported that *Acinetobacter baumannii* was frequently found in ICU COVID-19 patients (Sharifipour et al., 2020) with a high mortality rate and a more extended stay in the ICU (Ceparano et al., 2022). Meanwhile, there were high rates of *Staphylococcus aureus* isolated from respiratory tract samples in COVID-19 patients with ventilator-associated pneumonia and COVID-19 patients with community-acquired bacterial coinfections (Wu et al., 2022).

High prescription of antibiotics was a risk factor for antibiotic resistance. The prevalence of antibiotic prescription in COVID-19 patients was estimated as high as 74.6% (95% CI [68.3–80.0]) with an odd ratio increased in age years and mechanical ventilation used, but the estimated prevalence of bacterial coinfection in the review was low (8.6% (95% CI [4.7–15.2])) involving 31 studies (Langford et al., 2021). A review (Abu-Rub et al., 2021) also discovered a significant pattern of antibiotic prescription in the ICU settings for COVID-19 patients, along with much lower detection rates for bacterial identification. *Acinetobacter baumannii, Klebsiella pneumonia, Escherichia coli*, and *Pseudomonas aeruginosa* were the most frequently reported antibiotic-resistant Gram-negative bacteria during the COVID-19 pandemic. At the same time, *Staphylococcus aureus* and *Enterococcus faecium* were the most commonly encountered Gram-positive bacteria, indicating that antibiotic resistance was widespread during the pandemic (Sulayyim et al., 2022). The study also found that resistance of ESBL-producing *Escherichia coli* was 75%, 100% of *Staphylococcus aureus* and Coagulase-negative *Staphylococci* were resistant to methicillin, and, *Acinetobacter baumannii* and *Klebsiella pneumonia* were 91.7% and 76.6% respectively, carbapenem-resistant.

Particularly in COVID-19 patients with high rates of antimicrobial use and low rates of secondary or coinfection, the prevalence of multidrug-resistant organisms has rapidly risen (Lai et al., 2021). This review pooled the prevalence of antibiotic-resistant bacteria in ESBL (15.2%), CRAB (14.6%), CRPA (7.0%), MRSA (5.1%), CRE (5.0%), and VRE (1.3%). The difficulty differentiating between respiratory viral infection and bacterial coinfection led to the widespread use of broad-spectrum antibiotics. It has also been suggested that the severity of the clinical conditions in an environment with a high prevalence of bacteria that produce ESBL may have contributed to the widespread use of carbapenems in empirical therapy of COVID-19 infection (Rusic et al., 2021).

A review reported that the rate of *Acinetobacter baumannii* and *Klebsiella pneumoniae* carbapenem resistance significantly increased in 2020 compared to isolates from the pre-COVID-19 era, also a significant rise in polymyxin B resistance, particularly for *Klebsiella pneumoniae* isolates (Wu et al., 2022). According to several studies (Abdollahi et al., 2021; Eckardt et al., 2022; Shinohara et al., 2022), a CRAB strain had broken out in ICU COVID-19 wards due to cross-contamination between ICU equipment like ventilators, infusion pumps, and hemodialysis machines with the ICU equipment and central lines most likely contributed to the hospitalized COVID-19 patients’ five times higher prevalence of MRSA bacteremia (Aslam et al., 2022). MRSA and VRE were the most commonest detected among the Gram-positive antimicrobial resistance identified in Covid-19 patients (Kariyawasam et al., 2022). Therefore, coinfections with multidrug-resistant bacteria may result in COVID-19 patients having more extended hospital stays, extra costly treatment, and less desirable clinical outcomes (Senok et al., 2021).

This review provided the most recent pooled prevalence of bacterial coinfection and antibiotic resistance in hospitalized COVID-19 patients by including published publications from 2021 to 2022, as opposed to previous reviews that only had articles published in 2021. These findings should increase the consistency and accuracy of policymakers’ conclusions. However, the validity of these findings depended on the validity of the results of included studies.

As a limitation of this review, the regions subgroup may not represent the region since not all geographical parts were included in this study. For example, the African region was only represented by a study in Egypt and had the lowest prevalence of bacteria coinfection compared to other regions, with a greater number of included studies. Next, asymmetry funnel plots and significant Egger’s tests detected publication bias, indicating small-study effects. Therefore, the prevalence in this review may overestimate compared to the true value (van Aert, Wicherts & van Assen, 2019).

## Conclusions

Bacterial coinfection that includes bloodstream infection, secondary infection, and bacterial superinfection from published studies between January 2020 and September 2022 showed that the pooled prevalence was 27%. The subgroup analysis showed that the prevalence was highest in 2022, in the ICU and Europe, with the largest study participants. Meanwhile, *Acinetobacter baumannii* had the highest isolated bacteria prevalence compared to *Escherichia coli, Klebsiella pneumoniae, Pseudomonas aeruginosa*, and *Staphylococcus aureus*. The prevalence of antibiotic-resistant bacteria in hospitalized COVID-19 patients was 1.2%, and *ESBL-producing Enterobacteriaceae* contributed the highest prevalence compared to MRSA, VRE, CRE, CRAB, and CRPA. The findings of this review should be interpreted with caution due to their limitations. However, this review provided some evidence of bacterial coinfection in COVID-19 patients. This information might be valuable in developing an infection management and prevention strategy for COVID-19 patients.

## Supplemental Information

10.7717/peerj.15265/supp-1 Supplemental Information 1 Raw data indicating extraction of data from each included studies.

10.7717/peerj.15265/supp-2 Supplemental Information 2 Checklist of quality assessment for risk of bias.

10.7717/peerj.15265/supp-3 Supplemental Information 3 PRISMA checklist.

10.7717/peerj.15265/supp-4 Supplemental Information 4 Rationale.

## Additional Information and Declarations

### Data Availability

The following information was supplied regarding data availability: The raw data is available in the Supplemental File.
