# Supplementary material for: Bacterial coinfection and antibiotic resistance in hospitalized COVID-19 patients: a systematic review and meta-analysis
Source: PeerJ. 2023 Apr 26;11:e15265. doi: 10.7717/peerj.15265 (PMC10148641; doi:10.7717/peerj.15265)
Supplement: Supplemental Information 2 [file peerj-11-15265-s002.docx]

**S1 Table. Checklist of quality assessment for risk of bias**

| Author | Year | Was the sample frame appropriate to address the target population? | Were study participants sampled in an appropriate way? | Was the sample size adequate? | Were the study subjects and the setting described in detail? | Was a sample size justification, power description, or variance and effect estimates provided? | Were valid methods used for the identification of the condition? | Was the condition measured in a standard, reliable way for all participants? | Was there appropriate statistical analysis? | Was the response rate adequate, and if not, was the low response rate managed appropriately? | Total quality score | Risk of Bias: |
| --- | --- | --- | --- | --- | --- | --- | --- | --- | --- | --- | --- | --- |
| Ahmed et al | 2022 | yes | yes | yes | yes | no | yes | yes | yes | yes | 8 | low |
| Alqahtani et al | 2022 | yes | yes | yes | yes | no | yes | yes | yes | yes | 8 | low |
| Baggs et al | 2022 | yes | yes | yes | yes | no | yes | yes | yes | yes | 8 | low |
| Bahçe et al | 2022 | yes | yes | yes | yes | no | yes | yes | yes | yes | 8 | low |
| Baiou et al | 2021 | yes | yes | yes | yes | no | yes | yes | yes | yes | 8 | low |
| Baskaran et al | 2021 | yes | yes | yes | yes | no | yes | yes | yes | yes | 8 | low |
| Bazaid et al | 2022 | yes | yes | yes | yes | no | yes | yes | yes | yes | 8 | low |
| Bentivegna et al | 2021 | yes | yes | yes | yes | no | yes | yes | yes | yes | 8 | low |
| Bhaskaran | 2022 | yes | yes | yes | yes | no | yes | yes | yes | yes | 8 | low |
| Boorgula et al | 2022 | yes | yes | yes | yes | no | yes | yes | yes | yes | 8 | low |
| Ceccarelli et al | 2022 | yes | yes | yes | yes | no | yes | yes | yes | yes | 8 | low |
| Contou et al | 2020 | yes | yes | yes | yes | no | yes | yes | yes | yes | 8 | low |
| Copaja-Corzo et al | 2021 | yes | yes | yes | yes | yes | yes | yes | yes | yes | 9 | low |
| Cultrera et al | 2021 | yes | yes | yes | yes | no | yes | yes | yes | yes | 8 | low |
| da Costa et al | 2022 | yes | yes | yes | yes | no | yes | yes | yes | yes | 8 | low |
| Doubravská et al | 2022 | yes | yes | yes | yes | no | yes | yes | yes | yes | 8 | low |
| Floridia et al | 2022 | yes | yes | yes | yes | yes | yes | yes | yes | yes | 9 | low |
| Fontana et al | 2021 | yes | yes | yes | yes | no | yes | yes | yes | yes | 8 | low |
| Foschi et al | 2021 | yes | yes | yes | yes | no | yes | yes | yes | yes | 8 | low |
| Gysin et al | 2021 | yes | yes | yes | no | no | yes | yes | yes | yes | 7 | low |
| Jabir | 2022 | yes | yes | yes | yes | no | yes | yes | yes | yes | 8 | low |
| Jamnani et al | 2022 | yes | yes | yes | yes | no | yes | yes | yes | yes | 8 | low |
| Karataş et al | 2021 | yes | yes | yes | yes | no | yes | yes | yes | yes | 8 | low |
| Karruli et al | 2021 | yes | yes | yes | yes | no | yes | yes | yes | yes | 8 | low |
| Khurana et al | 2021 | yes | yes | yes | yes | no | yes | yes | yes | yes | 8 | low |
| Kubin et al | 2021 | yes | yes | yes | yes | no | yes | yes | yes | yes | 8 | low |
| Li et al | 2020 | yes | yes | yes | yes | no | yes | yes | yes | yes | 8 | low |
| Lingscheid et al | 2022 | yes | yes | yes | yes | no | yes | yes | yes | yes | 8 | low |
| Mahmoudi | 2020 | yes | yes | yes | yes | no | yes | yes | yes | yes | 8 | low |
| Mazzariol et al | 2021 | yes | yes | yes | yes | no | yes | yes | yes | yes | 8 | low |
| Moradi et al | 2021 | yes | yes | yes | yes | no | yes | yes | yes | yes | 8 | low |
| Nori et al | 2021 | yes | yes | yes | yes | no | yes | yes | yes | yes | 8 | low |
| Palanisamy et al | 2021 | yes | yes | yes | yes | no | yes | yes | yes | yes | 8 | low |
| Posteraro et al | 2021 | yes | yes | yes | yes | no | yes | yes | yes | yes | 8 | low |
| Pourajam et al | 2022 | yes | yes | yes | yes | no | yes | yes | yes | yes | 8 | low |
| Protonotariou et al | 2021 | yes | yes | yes | yes | no | yes | yes | yes | yes | 8 | low |
| Ramadan et al | 2020 | yes | yes | yes | yes | no | yes | yes | yes | yes | 8 | low |
| Rao et al | 2022 | yes | yes | yes | yes | no | yes | yes | yes | yes | 8 | low |
| Rizvi et al | 2022 | yes | yes | yes | no | no | yes | yes | yes | yes | 7 | low |
| Said et al | 2022 | yes | yes | yes | yes | no | yes | yes | yes | yes | 8 | low |
| Sathyakamala et al | 2022 | yes | yes | yes | yes | no | yes | yes | yes | yes | 8 | low |
| Sharifipour et al | 2020 | yes | yes | yes | yes | no | yes | yes | yes | yes | 8 | low |
| Sharma et al | 2021 | yes | yes | yes | yes | no | yes | yes | yes | yes | 8 | low |
| Sinto et al | 2022 | yes | yes | yes | yes | no | yes | yes | yes | yes | 8 | low |
| Son et al | 2021 | yes | yes | yes | yes | no | yes | yes | yes | yes | 8 | low |
| Suarez-de-la-Rica et al | 2021 | yes | yes | yes | yes | no | yes | yes | yes | yes | 8 | low |
| Temperoni et al | 2021 | yes | yes | yes | yes | no | yes | yes | yes | yes | 8 | low |
| Vijay et al | 2021 | yes | yes | yes | yes | no | yes | yes | yes | yes | 8 | low |
| Zeshan et al | 2022 | yes | yes | yes | yes | no | yes | yes | yes | yes | 8 | low |
