# Supplementary material for: Bacterial coinfection and antibiotic resistance in hospitalized COVID-19 patients: a systematic review and meta-analysis
Source: PeerJ. 2023 Apr 26;11:e15265. doi: 10.7717/peerj.15265 (PMC10148641; doi:10.7717/peerj.15265)
Supplement: Supplemental Information 4 [file peerj-11-15265-s004.docx]

1. The rationale for conducting the systematic review / meta-analysis

This systematic review and meta-analysis aimed to reveal the pooled prevalence of bacterial coinfection and antibiotic resistance in hospitalized COVID-19 patients. This information integrates existing information to determine if scientific conclusions are consistent and generalizable across populations and situations or whether findings significantly differ by subgroup. It also aids healthcare shareholders in making decisions because the pooled prevalence increases the power and precision of effect estimations.

1. The contribution that it makes to knowledge in light of previously published related reports, including other meta-analyses and systematic reviews.

This review provided the most recent pooled prevalence of bacterial coinfection and antibiotic resistance in hospitalized COVID-19 patients by including published publications from 2021 to 2022, as opposed to previous reviews that only had articles published in 2021. These findings should increase the consistency and accuracy of policymakers’ conclusions.
